# Plasma microRNA-210 is associated with VEGF-A and EphrinA3 and relates to coronary collateral circulation in patients with coronary heart disease: a cross-sectional study

**DOI:** 10.1186/s12872-025-05013-y

**Published:** 2025-07-28

**Authors:** Ning Zhao, Kun Na, Wei Sun, Henghe Shi, Xiaolin Zhang, Bin Liu, Yaling Han

**Affiliations:** 1 State Key Laboratory of Frigid Zone Cardiovascular Disease, Cardiovascular Research Institute, Department of Cardiology, General Hospital of Northern Theater Command, Shenyang, China; 2 Department of Cardiology, Second Norman Bethune Hospital of Jilin University, No. 218 Ziqiang Street, Changchun, China

**Keywords:** Coronary heart disease, Collateral circulation, MicroRNA-210, EphrinA3, VEGF-A

## Abstract

**Background:**

This study explored the interrelationships among vascular endothelial growth factor A (VEGF-A), microRNA-210 (miR-210), and EphrinA3 in the plasma of patients with coronary heart disease (CHD), and their collective influence on coronary collateral circulation (CCC) development.

**Methods:**

We enrolled 253 patients with ≥ 90% stenosis in at least one coronary artery, stratified into good CCC (*n* = 99) and poor CCC (*n* = 154) groups according to the Rentrop grading system. Plasma concentrations of miR-210, VEGF-A, and EphrinA3 were quantified via qRT-PCR and ELISA. The associations between these biomarkers and CCC status were evaluated through correlation analysis, multivariate regression, and mediation analysis.

**Results:**

Good CCC patients demonstrated significantly elevated plasma miR-210 (1.936 [1.099–4.118] vs. 1.272 [0.792–2.081], *p* < 0.001) and VEGF-A levels (3119.655 ± 850.995 vs. 2910.440 ± 713.218 pg/mL, *p* = 0.038), alongside reduced EphrinA3 levels (529.594 ± 143.037 vs. 584.657 ± 127.182 pg/mL, *p* = 0.002) compared to poor CCC patients. ROC analysis revealed AUCs of 0.656 (95% CI: 0.589–0.724) for miR-210, 0.563 (95% CI: 0.489–0.638) for VEGF-A, and 0.632 (95% CI: 0.560–0.705) for EphrinA3, which improved to 0.747, 0.696, and 0.744 respectively after adjustment for confounders. In fully adjusted multivariate models, miR-210 maintained a robust positive association with good CCC (OR: 1.558, 95% CI: 1.257–1.931, *p* < 0.001), with its highest tertile conferring 4.58-fold increased odds compared to the lowest tertile. Conversely, EphrinA3 exhibited a significant negative association (OR: 0.993, 95% CI: 0.990–0.997, *p* < 0.001), with its highest tertile linked to 79.4% reduced odds of good CCC. VEGF-A showed a modest association (OR: 1.001, *p* = 0.043). Notably, mediation analysis revealed that miR-210 functions as a pivotal intermediary in pathways connecting both VEGF-A and EphrinA3 to CCC formation, mediating 77.18% and 49.90% of their respective effects.

**Conclusions:**

Plasma miR-210 levels exhibit a strong association with coronary collateral circulation development and represent a promising biomarker for CCC formation in patients with severe coronary stenosis. The influence of VEGF-A and EphrinA3 on CCC formation appears to be predominantly mediated through miR-210, highlighting its central role in coronary collateralization pathways.

**Supplementary Information:**

The online version contains supplementary material available at 10.1186/s12872-025-05013-y.

## Background

Coronary collateral circulation (CCC) serves as a physiological adaptive mechanism responding to significant stenosis of the coronary arteries or myocardial ischemia in patients with coronary heart disease (CHD) [1]. Well-developed CCC provides additional blood supply to ischemic myocardium, improving outcomes and quality of life [2], with numerous studies demonstrating lower mortality rates and reduced adverse cardiac events following myocardial infarction [3–6]. Additionally, retrograde percutaneous coronary intervention (PCI) through coronary collaterals for chronic total occlusion lesions can enhance the success rate of interventional treatment [7–10]. CCC development occurs through angiogenesis and arteriogenesis [11], influenced by clinical characteristics and various biochemical factors [12–22], with hypoxia playing a pivotal role as evidenced by rich coronary collaterals in patients with sleep apnea [23], high-altitude residents [24], and experimental models showing hypoxia alone inducing collateral vessel formation [25, 26].

Molecular mediators orchestrate hypoxia-driven collateralization through complex interactions. Vascular endothelial growth factor A (VEGF-A), the principal angiogenic factor, contributes significantly to collateral development [25, 27–30], with VEGF-A knockout inhibiting CCC formation in hypoxic environments [26]. MicroRNA-210 (miR-210), the predominant hypoxia-inducible miRNA [31, 32], is upregulated in hypoxic conditions [33, 34] and modulates VEGF-A expression [35–38] while promoting endothelial cell survival, migration, and angiogenic capacity [38–42]. Notably, miR-210 downregulates EphrinA3, one of its targets, thereby potentially enhancing angiogenesis [39, 43–45]. Despite these individual roles, the functional hierarchy and interrelationships among these molecules in human coronary collateralization remain poorly understood.

This study aimed to elucidate the interrelationships among miR-210, VEGF-A, and EphrinA3 and their association with CCC development in patients with significant coronary stenosis. Specifically, we sought to determine whether miR-210 functions as a central mediator between VEGF-A, EphrinA3, and CCC formation, and to evaluate whether plasma miR-210 levels could serve as a potential biomarker for assessing coronary collateral formation in these patients.

## Methods

### Study population

Sample size was determined using the power.roc.test function in the pROC package (R software) for ROC curve power analysis during the study design phase. This cross-sectional study ultimately enrolled 253 participants (161 males, 92 females) with at least one epicardial coronary artery stenosis ≥ 90%, recruited between November 2016 and November 2017 at the Third Catheterization Laboratory of the Second Norman Bethune Hospital of Jilin University.

Exclusion criteria comprised: (1) age < 18 or > 90 years; (2) acute myocardial infarction within the previous 3 months; (3) prior percutaneous coronary intervention or coronary artery bypass grafting; (4) concomitant valvular heart disease; (5) heart failure; (6) other coronary artery diseases or structural anomalies; (7) vascular comorbidities including peripheral vascular disease, renal artery stenosis, cerebral infarction, cerebral hemorrhage, or other arterial diseases; and (8) significant hepatic or renal dysfunction, hematologic disorders, systemic immune diseases, malignancies, acute or chronic infections, or chronic respiratory diseases. Fourteen additional patients were excluded due to incomplete data or blood sample inadequacies. The study protocol received approval from the Ethics Review Committee at the Second Norman Bethune Hospital of Jilin University and was conducted in accordance with the Declaration of Helsinki guidelines. All participants provided written informed consent. Clinical trial number: not applicable.

### Clinical data collection

Following informed consent, comprehensive baseline data were collected from all participants, including demographic information, CHD risk factors, biochemical parameters, and current medications. Hypertension was defined as a systolic blood pressure (SBP) ≥ 140 mmHg and/or a diastolic blood pressure (DBP) ≥ 90 mmHg, or the use of antihypertensive medications to control blood pressure. Diabetes was defined according to the diagnostic criteria of the American Diabetes Association. The statins used in this study included atorvastatin, rosuvastatin, simvastatin, and pitavastatin. All data were systematically recorded in standardized case report forms and subsequently verified for accuracy and completeness by trained research personnel.

### Coronary angiography and collateral scoring

During the angiography, multi-positional projections were employed, with each position maintained for at least five cardiac cycles to ensure that the contrast agent could fully perfuse and delineate coronary artery lesions, as well as adequately visualize collateral vessels. The results were interpreted by a minimum of two interventional cardiologists. If there are scoring discrepancies, a third expert will participate in the discussion to reach a consensus. All raters are blinded to the clinical information. The degree of coronary stenosis was assessed using the Gensini score [46]. The severity of single vessel lesions was evaluated first, followed by an assessment of different segments of the coronary arteries. The score for each vessel was calculated as the score of that vessel multiplied by a scoring coefficient, with the final score being the cumulative total of the scores for all three vessels (Table S1). Based on the Rentrop grading system [47], patients in the experimental group were categorized into a good CCC group and a poor CCC group. A Rentrop grading of ≥ 2 was indicative of good CCC, while a Rentrop grading of <2 was indicative of poor CCC.

### Plasma sample collection

During the angiography procedure, prior to administration, 5 mL of arterial blood was drawn through the sheath into an ethylenediaminetetraacetic acid-coated anticoagulant tube. The tube was gently inverted 2–3 times and then allowed to stand undisturbed. The sample underwent centrifugation at a low temperature of 4 °C for 10 min at a speed of 4000 revolutions per min (rpm) within a 12-hour period. The plasma supernatant was then meticulously gathered and preserved in a freezer set to −80 °C.

### Quantitative reverse transcription polymerase chain reaction (qRT-PCR) evaluation of miR-210 expression

The evaluation of miR-210 expression was carried out utilizing qRT-PCR. Thaw cryopreserved plasma at 4 °C, transfer 250 µl to an Rnase-free tube, add cel-mir39 (50 pmol/L), and Trizol (Invitrogen, United States) for RNA extraction. Store samples at −80 °C and assess purity and integrity with Nanodrop and Agilent Bioanalyzer before testing. The total RNA underwent reverse transcription employing the miScript reverse transcription kit (TRANS GEN, China), adhering to the instructions provided by the manufacturer. Specific miRNA assays were utilized to ascertain gene expression levels. The quantitative polymerase chain reaction (qPCR) reactions were performed in a total volume of 20 µl, which comprised 2 µl of the reverse-transcribed products, 1.0 µl of the 20× Micro RNA assay primer, and 10 µl of 2× Universal PCR Master Mix, along with nuclease-free H2O to achieve the final volume. Amplification was monitored using the Viia7 qPCR instrument (Applied Biosystems), which was set to initiate with a 1-minute step at 95˚C, followed by 40 cycles that consisted of 20 s at 95˚C and 34 s at 60˚C. The relative levels of gene expression were evaluated using the formula 2^-ΔCT, where ΔCT is derived from CT (target gene) - CT (control), with normalization conducted with cel-miR-39. All miR-210 and cel-miR-39 samples were analyzed in a single assay, and all qRT-PCR reactions were repeated three times, and the average values were used for analysis. The amplification primer sequences are listed in Table S2.

### Enzyme-linked immunosorbent assay (ELISA) evaluation of VEGF-A and EphrinA3 expression

Plasma concentrations of VEGF-A and EphrinA3 were assessed utilizing ELISA kits for human VEGF-A and EphrinA3 (R&D, United States) in accordance with the guidelines provided by the manufacturer. The absorbance was measured at a primary wavelength of 450 nm. All ELISA experiments were performed in duplicate and the average values were used for analysis.

### Statistical analyses

Categorical variables are presented as frequencies and percentages, compared using chi-squared or Fisher’s exact tests. Continuous variables were tested for normality using Shapiro-Wilk test. Normally distributed variables are expressed as mean ± SD and compared using independent t-tests; non-normally distributed variables as median (IQR) and compared using Mann-Whitney U tests. Correlations were assessed using Spearman’s coefficient and Restricted Cubic Spline (RCS) analysis for nonlinear relationships. Receiver operating characteristic (ROC) curves were constructed to evaluate the area under the curve (AUC) for diagnostic performance of plasma biomarkers for predicting good CCC, both unadjusted and adjusted for confounders. Associations between biomarkers and CCC status were examined using crude and adjusted logistic regression models. Mediation analyses using bootstrap method (1000 repetitions) with generalized linear models (probit link) assessed miR-210’s mediating role between VEGF-A/EphrinA3 and CCC formation. Subgroup analyses evaluated associations across clinical categories (sex, estimated glomerular filtration rate [eGFR], age, body mass index [BMI], diabetes, hypertension, smoking) with interaction tests to assess heterogeneity among subgroups. Listwise deletion were used to handle missing data. All statistical tests were two-tailed, with *p* < 0.05 considered significant. Analyses were performed using R software (version 4.2.0).

## Results

### Baseline characteristics

**Fig. 1:**
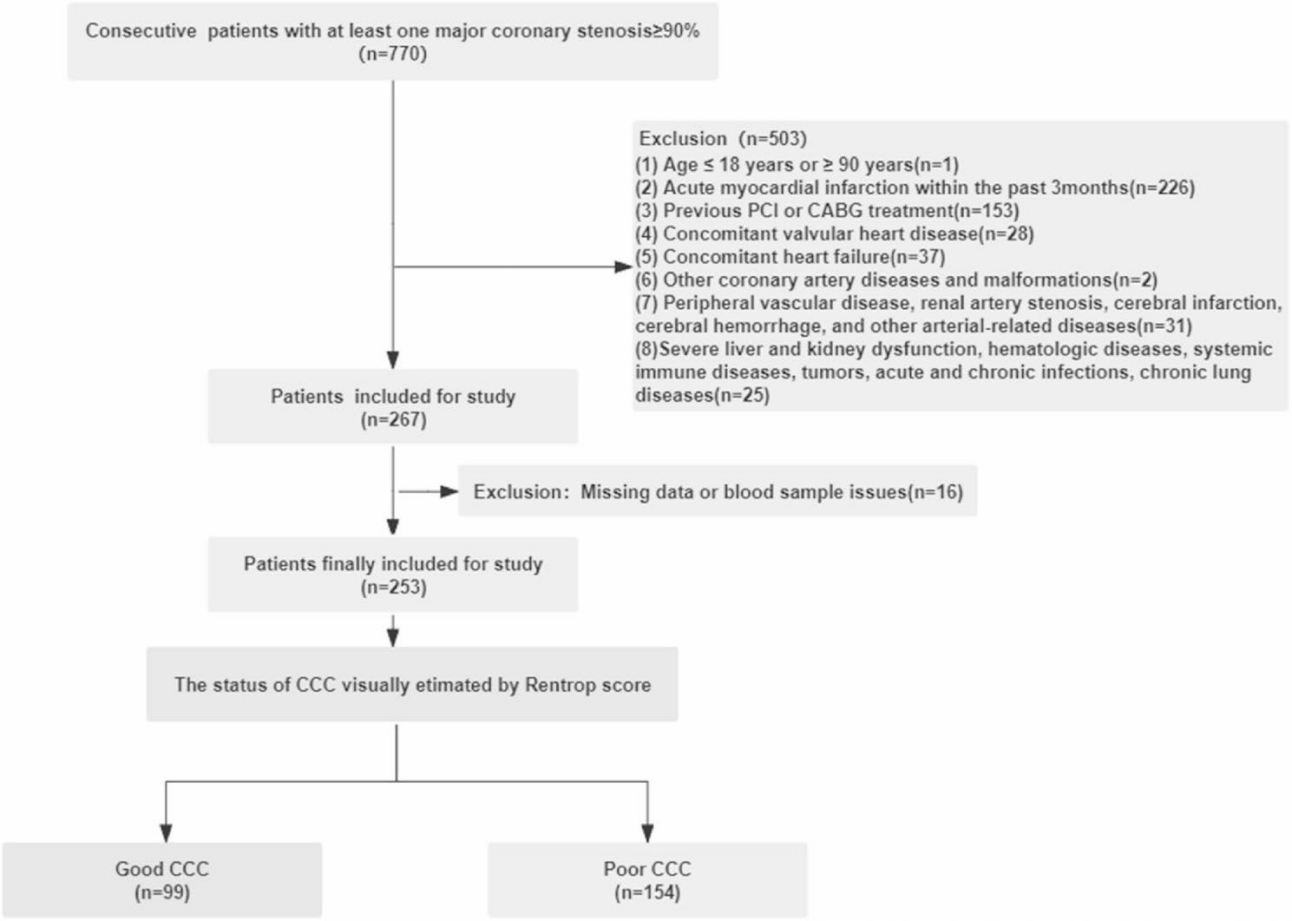
Flowchart of patient enrollment. CCC, coronary collateral circulation; CABG, Coronary Artery Bypass Grafting; PCI, percutaneous coronary intervention

The study included 154 individuals with poor CCC and 99 with good CCC (Fig. 1). Both groups showed similar demographic, clinical, and biochemical parameters, including age, gender, blood pressure, heart rates, smoking habits, most blood parameters, and medication use (Table 1). However, the good CCC group demonstrated significantly higher Gensini scores (108.00 [85.000–132.000] vs. 80.000 [49.000–112.000], *p* < 0.001), lower HDL-C levels (1.043 ± 0.260 vs. 1.176 ± 0.280 mmol/L, *p* < 0.001), higher BMI (27.446 ± 16.924 vs. 24.471 ± 2.972, *p* = 0.045), increased diabetes prevalence (42.11% vs. 27.74%, *p* = 0.023), elevated serum uric acid (341.968 ± 93.717 vs. 317.860 ± 84.231, *p* = 0.043), and reduced LVEF (58.726 ± 10.218 vs. 62.233 ± 7.827, *p* = 0.004).

**Table 1:** Baseline clinical characteristics in patients with poor and good CCC

	Overall (*n* = 253)	Good CCC (*n* = 99)	Poor CCC (*n* = 154)	*P*-value
Clinical				
Age, years	62.228 ± 8.646	62.189 ± 8.600	62.255 ± 8.709	0.955
Male, *n* (%)	161 (69.40%)	69 (72.63%)	92 (67.15%)	0.373
BMI, Kg/m^2^	25.642 ± 10.931	27.446 ± 16.924	24.471 ± 2.972	0.045
Cigarette smoking, *n* (%)	96 (41.56%)	46 (48.42%)	50 (36.77%)	0.077
Heart rates, t/m	74.780 ± 11.970	75.000 ± 13.160	74.628 ± 11.118	0.816
Systolic blood pressure, mmHg	136.207 ± 20.145	135.505 ± 17.739	136.693 ± 21.707	0.660
Diastolic blood pressure, mmHg	83.522 ± 10.958	83.368 ± 10.758	83.628 ± 11.134	0.860
Hypertension, *n* (%)	135 (58.19%)	57 (60.00%)	78 (56.93%)	0.642
Diabetes, n (%)	78 (33.62%)	40 (42.11%)	38 (27.74%)	0.023
Laboratory				
Fasting blood glucose, mmol/L	6.505 ± 2.111	6.628 ± 2.023	6.422 ± 2.173	0.470
Homocysteine, mmol/L	20.217 ± 10.830	20.931 ± 11.645	19.700 ± 10.217	0.421
Free fatty acids	0.601 ± 0.289	0.584 ± 0.259	0.613 ± 0.310	0.478
Triglyceride, mmol/L	1.898 ± 1.589	1.942 ± 1.483	1.868 ± 1.665	0.742
Total cholesterol, mmol/L	4.830 ± 1.240	4.833 ± 1.130	4.829 ± 1.315	0.982
HDL cholesterol, mmol/L	1.123 ± 0.260	1.043 ± 0.204	1.176 ± 0.280	< 0.001
LDL cholesterol, mmol/L	2.712 ± 0.940	2.790 ± 0.923	2.658 ± 0.951	0.313
Lipoprotein (a), g/L	125.050 ± 36.706	119.775 ± 35.951	128.843 ± 36.921	0.079
Apolipoprotein B, g/L	88.479 ± 28.810	90.310 ± 22.864	87.162 ± 32.450	0.438
Apolipoprotein A, g/L	258.987 ± 218.637	279.275 ± 249.476	244.279 ± 193.009	0.257
Serum uric acid, µmol/L	327.713 ± 88.829	341.968 ± 93.717	317.860 ± 84.231	0.043
eGFR, ml/min/1.73m^2^	51.427 ± 12.295	50.190 ± 12.712	52.297 ± 11.964	0.201
hsCRP, mg/L	1.810 (0.820–4.430)	2.300 (0.950–4.100)	1.395 (0.708–4.867)	0.127
WBC	7.061 ± 1.761	7.299 ± 1.771	6.899 ± 1.741	0.091
Platelet, 10^^^9/L	221.236 ± 52.012	218.186 ± 52.880	223.322 ± 51.502	0.464
miR-210, Fold Change	1.544 (0.910–2.776)	1.936 (1.099–4.118)	1.272 (0.792–2.081)	< 0.001
VEGF-A, pg/mL	2994.980 ± 777.005	3119.655 ± 850.995	2910.440 ± 713.218	0.038
EphrinA3, pg/mL	562.407 ± 136.246	529.594 ± 143.037	584.657 ± 127.182	0.002
Cardiac systolic function				
LVEF, %	60.815 ± 9.017	58.726 ± 10.218	62.233 ± 7.827	0.004
Severity of CHD				
Gensini score	80.000 (49.000-112.000)	108.000 (85.000-132.000)	80.000 (49.000-112.000)	< 0.001
Medication, *n* (%)				
Statins	132 (56.90%)	54 (56.84%)	78 (56.93%)	0.989
ACE inhibitors/ARBs	60 (25.86%)	28 (29.47%)	32 (23.36%)	0.295

Values are given as mean ± standard deviation (SD), median (25th ~ 75th percentile) or number (percentage). For normally distributed data, an independent samples t-test is used. For non-normally distributed data, the Mann-Whitney U test is employed. For categorical variables, the chi-square test or Fisher’s exact test is used

*CCC* coronary collateral circulation, *BMI* body mass index, *eGFR* estimated glomerular filtration rate, *LDL* low-density lipoprotein, *HDL* high-density lipoprotein,* hsCRP* high-sensitivity C-reactive protein, *WBC* white blood cell, *CHD* coronary heart disease, *LVEF* left ventricular ejection fraction, *ACE* angiotensin-converting enzyme

### Plasma VEGF-A, miR-210, and EphrinA3 and their correlations in CHD patients

**Fig. 2:**
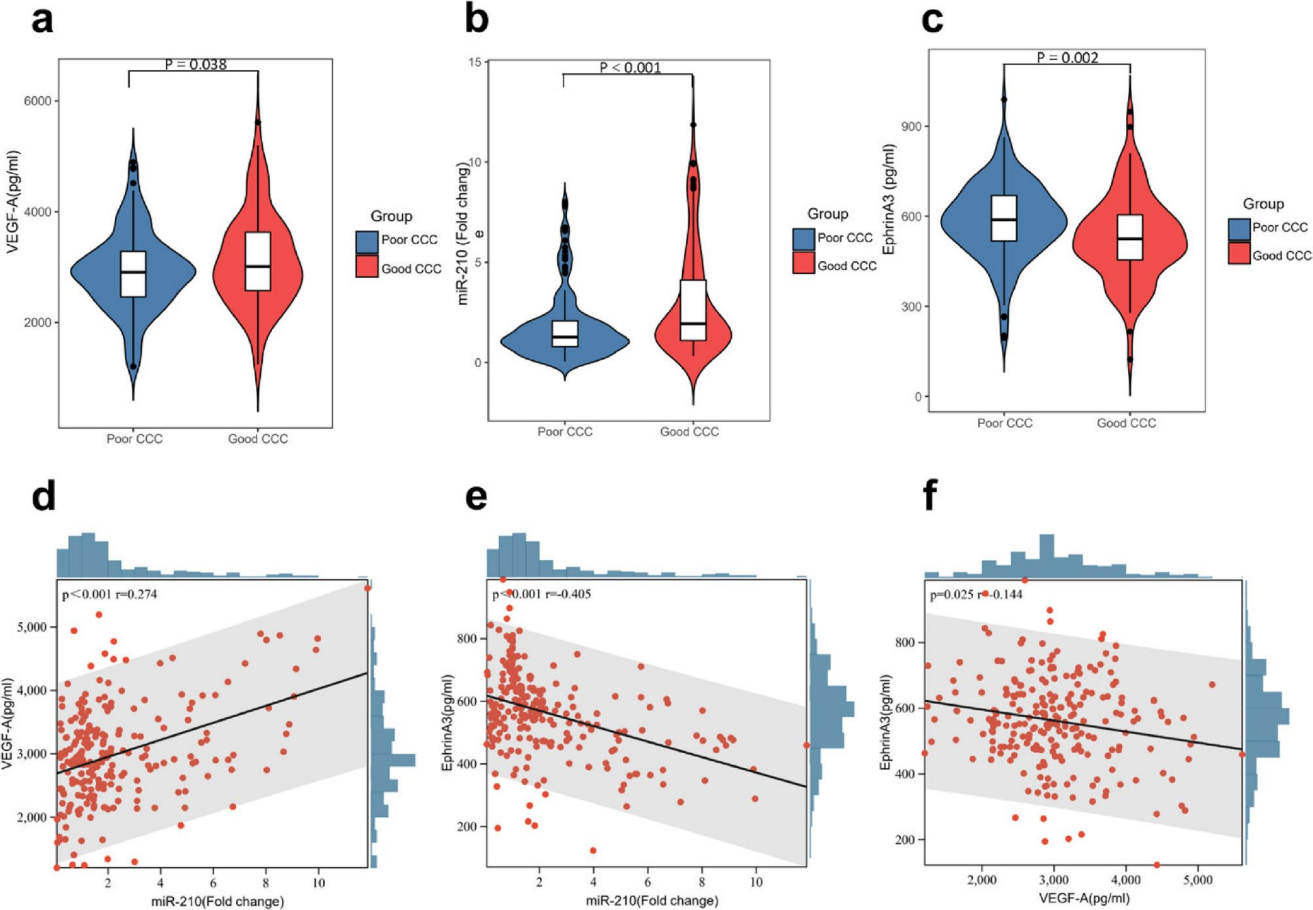
Plasma VEGF-A, miR-210 and EphrinA3 and their correlations in patients with CHD. **a**-**c** The plasma level of VEGF-A, miR-210 and EphrinA3 in CHD patients. **d**-**f** The linear correlation analysis between miR-210 and VEGF-A, miR-210 and EphrinA3, VEGF-A and EphrinA3. CHD, coronary heart disease; CCC, coronary collateral circulation

VEGF-A and miR-210 levels were significantly higher in the good CCC group (3119.655 ± 850.995 vs. 2910.440 ± 713.218 pg/mL, *p* = 0.038; and 1.936 [1.099–4.118] vs. 1.272 [0.792–2.081], *p* < 0.001, respectively), while EphrinA3 levels were significantly lower (529.594 ± 143.037 vs. 584.657 ± 127.182 pg/mL, *p* = 0.002) (Fig. 2a-c; Table 1). Correlation analysis revealed a positive correlation between miR-210 and VEGF-A (*r* = 0.274, *p* < 0.001), a negative correlation between miR-210 and EphrinA3 (*r*=−0.405, *p* < 0.001), and a negative correlation between EphrinA3 and VEGF-A (*r*=−0.144, *p* = 0.025) (Fig. 2d-f). Additionally, correlation analyses between metabolic parameters (BMI, diabetes, HDL-C, serum uric acid) and plasma VEGF-A, miR-210, and EphrinA3 levels revealed no significant correlations (Figure S1).

### Associations between plasma VEGF-A, miR-210, EphrinA3 and collateral formation

**Fig. 3:**
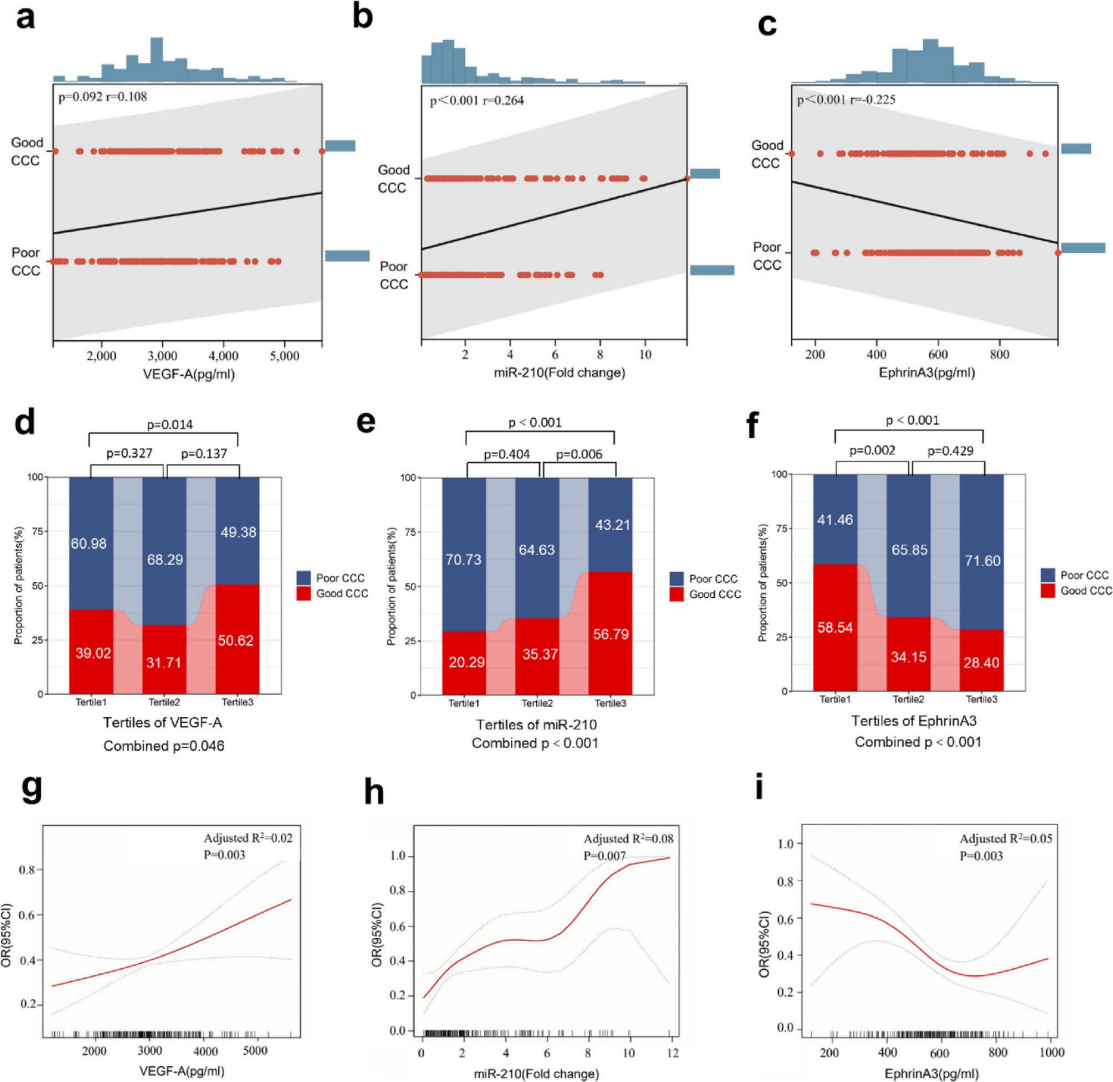
Plasma VEGF-A, miR-210, EphrinA3 and collateral formation. **a**-**c** The correlation analysis between VEGF-A, miR-210, EphrinA3 and Good CCC. **d**-**f** The proportion of good CCC or poor CCC from the lowest tertile to the highest tertile of VEGF-A, miR-210 and EphrinA3. **g**-**i** The nonlinear correlation analysis between miR-210, VEGF-A, EphrinA3 and Good CCC. OR, odds ratio; CCC, coronary collateral circulation

Analysis of biomarkers and CCC level showed no significant correlation with VEGF-A (*r* = 0.108, *p* = 0.108), but positive correlation with miR-210 (*r* = 0.264, *p* < 0.001) and negative correlation with EphrinA3 (*r*=−0.225, *p* < 0.001) (Fig. 3a-c). When stratified by tertiles, the proportion of good CCC across increasing VEGF-A tertiles showed no consistent trend (39.02%, 31.71%, 50.62%), though an overall significant difference was observed (*p* = 0.046), with significance between Tertile 3 and Tertile 1 (*p* = 0.014) (Fig. 3d). For miR-210, the proportion of good CCC increased with ascending tertiles (20.29%, 35.37%, 56.79%; *p* < 0.001), with significant differences between Tertile 3 and Tertile 2 (*p* = 0.006), and Tertile 3 and Tertile 1 (*p* < 0.001) (Fig. 3e). Conversely, the proportion of good CCC decreased with increasing EphrinA3 tertiles (58.54%, 34.15%, 28.40%; *p* < 0.001), with significant differences between Tertile 2 and Tertile 1 (*p* = 0.002), and Tertile 3 and Tertile 1 (*p* < 0.001) (Fig. 3f).

RCS analysis demonstrated that the likelihood of good CCC formation increased nonlinearly with elevated miR-210 levels, showed subtle nonlinear relationship with VEGF-A, and decreased nonlinearly with elevated EphrinA3 levels (Fig. 3g-i).

Sensitivity analyses were conducted across various subgroups based on gender, eGFR levels (< 60 or ≥ 60 mL/min/1.73 m²), age (< 65 or ≥ 65 years), BMI (< 25 or ≥ 25 kg/m²), and presence of diabetes, hypertension, and smoking. Plasma miR-210 consistently demonstrated diagnostic value for effective CCC formation across all subgroups, with ORs ranging from 1.218 to 1.519 (P interaction ≥ 0.2861) (Figure S2).

### Predictive capabilities of VEGF-A, miR-210, and EphrinA3 on good CCC

**Fig. 4:**
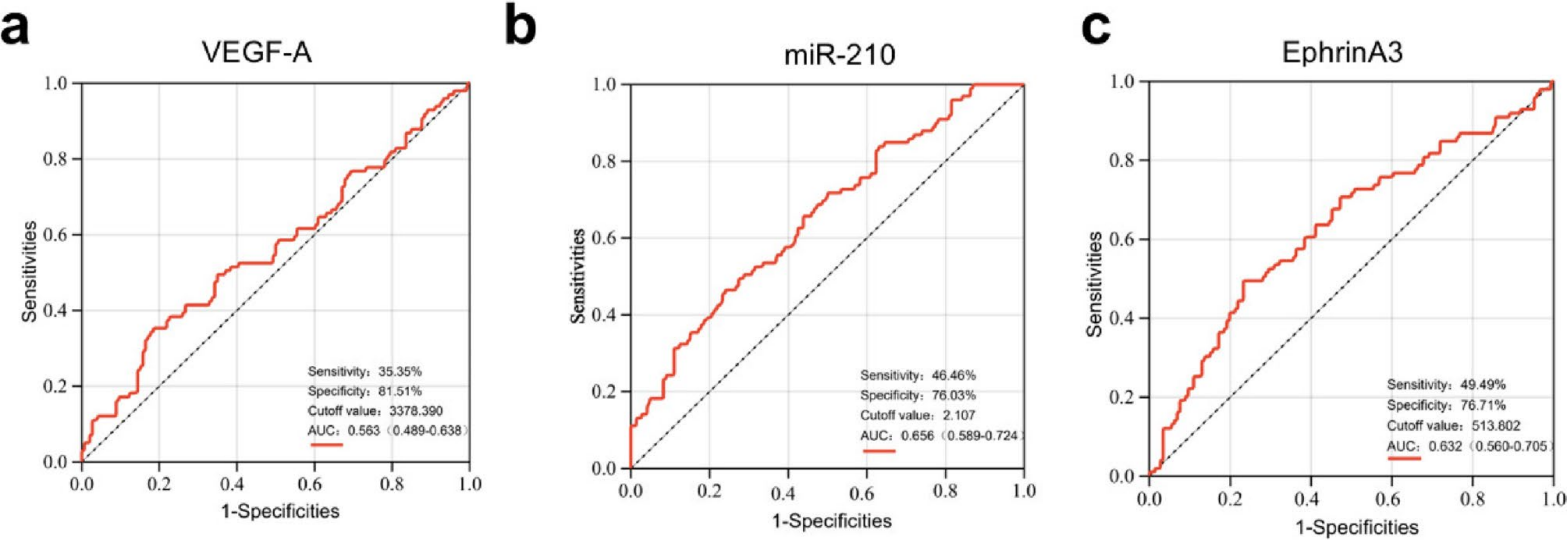
Receiver-operating characteristic curve analysis for identifying Good CCC. **a**-**c** ROC curves evaluating the diagnostic performance of VEGF-A, miR-210 and EphrinA3 associated with good CCC. CCC, coronary collateral circulation; ROC, receiver operating characteristic; AUC, area under the curve

ROC curve analysis showed that the AUC for VEGF-A was 0.563 (95% CI: 0.489–0.638), with 35.35% sensitivity, 81.51% specificity, and a cutoff value of 3378.390 pg/mL (Fig. 4a). The AUC for miR-210 was 0.656 (95% CI: 0.589–0.724), with 46.46% sensitivity, 76.03% specificity, and a cutoff value of 2.107 (Fig. 4b). For EphrinA3, the AUC was 0.632 (95% CI: 0.560–0.705), with 49.49% sensitivity, 76.71% specificity, and a cutoff value of 513.802 pg/mL (Fig. 4c). After adjusting for confounding variables (age, sex, HDL-C, and LVEF), the AUC improved significantly: VEGF-A increased from 0.563 to 0.696 (*p* = 0.003), miR-210 from 0.656 to 0.747 (*p* = 0.041), and EphrinA3 from 0.632 to 0.744 (*p* = 0.011), as detailed in Table 2.

**Table 2:** ROC curve analysis adjusted for confounding factors

Variable	VEGF-A			miR-210			EphrinA3		
	Crude Model	Adjust Model	*P* (compare)	Crude Model	Adjust Model	*P* (compare)	Crude Model	Adjust Model	*P* (compare)
Sensitivity%	35.35	58.89	0.003	46.46	76.67	0.041	49.49	54.44	0.011
Specificity%	81.51	72.95		76.03	61.48		76.71	83.61	
AUC	0.563 (0.489, 0.638)	0.696 (0.625, 0.767)		0.656 (0.589, 0.724)	0.747 (0.682, 0.813)		0.632 (0.560, 0.705)	0.744 (0.677, 0.810)	

Crude model adjust for: None;

Adjust model 1 adjust for: Age; Male; LVEF; HDL cholesterol

*ROC* receiver operating characteristic, *AUC* area under the curve

### Multivariate regression analysis for the effect of VEGF-A, miR-210 and EphrinA3 on the formation of good CCC

To evaluate the independent associations of plasma biomarkers with good CCC formation while controlling for confounding variables, multivariate regression analysis was performed (Table 3). Three models were constructed: Crude Model (unadjusted), Model 1 (adjusted for age, sex, and smoking), and Model 2 (further adjusted for LVEF, HDL cholesterol, BMI, and diabetes).

**Table 3:** Multivariative regression for effect of plasma VEGF-A, miR-210 and EphrinA3 on good CCC

Variable	Crude Model			Multivariable-Adjusted Model 1			Multivariable-Adjusted Model 2		
	OR (95%)	*P* value	*P* for trend	OR (95%)	*P* value	*P* for trend	OR (95%)	*P* value	*P* for trend
VEGF-A									
Total (continuous)	1.000 (1.000, 1.001)	0.123	-	1.000 (1.000, 1.001)	0.207	-	1.001 (1.000, 1.001)	0.043	-
Tertile1 (< 2655.7)	1		< 0.001	1		< 0.001	1		< 0.001
Tertile2 (2655.7 ~ 3235.2)	0.739 (0.388, 1.406)	0.356		0.673 (0.341, 1.329)	0.254		0.835 (0.394, 1.767)	0.637	
Tertile3 (> 3235.2)	1.562 (0.841, 2.904)	0.158		1.434 (0.749, 2.745)	0.277		2.082 (0.985, 4.401)	0.055	
miR-210									
Total (continuous)	1.385 (1.160, 1.653)	< 0.001	-	1.437 (1.190, 1.734)	< 0.001	-	1.558 (1.257, 1.931)	< 0.001	-
Tertile1 (< 1.08)	1		< 0.001	1		< 0.001	1		< 0.001
Tertile2 (1.08 ~ 2.10)	1.348 (0.698, 2.602)	0.374		1.284 (0.639, 2.579)	0.482		1.441 (0.657, 3.160)	0.362	
Tertile3 (> 2.10)	3.088 (1.620, 5.888)	< 0.001		3.414 (1.718, 6.784)	< 0.001		4.580 (2.088, 10.043)	< 0.001	
EphrinA3									
Total (continuous)	0.995 (0.992, 0.998)	< 0.001	-	0.995 (0.992, 0.998)	< 0.001	-	0.993 (0.990, 0.997)	< 0.001	-
Tertile1 (< 512.2)	1		< 0.001	1		< 0.001	1		< 0.001
Tertile2 (512.2 ~ 613.2)	0.354 (0.187, 0.670)	0.001		0.383 (0.194, 0.756)	0.006		0.374 (0.174, 0.803)	0.012	
Tertile3 (> 613.2)	0.293 (0.153, 0.560)	< 0.001		0.283 (0.143, 0.557)	< 0.001		0.206 (0.095, 0.446)	< 0.001	

Crude model adjust for: None;

Adjusted model 1 adjust for: Age; Male; Smoking;

Adjusted model 2 adjust for: Age; Male; Smoking; LVEF; HDL cholesterol; BMI; Diabetes

*CCC* coronary collateral circulation, *LVEF* left ventricular ejection fraction, *HDL* high-density lipoprotein, *BMI* body mass index, *OR* odds ratio

VEGF-A showed no significant association with good CCC in either the Crude Model (OR: 1.000 [1.000, 1.001], *p* = 0.123) or Model 1 (OR: 1.000 [1.000, 1.001], *p* = 0.207). Despite reaching statistical significance in Model 2 (OR: 1.001 [1.000–1.001], *p* = 0.043), the minimal OR value indicates a clinically negligible effect. Analysis by VEGF-A tertiles confirmed no significant associations. In contrast, miR-210 demonstrated a robust positive association with good CCC across all models, strengthening after adjustment: Crude Model (OR: 1.385 [1.160, 1.653], *p* < 0.001), Model 1 (OR: 1.437 [1.190, 1.734], *p* < 0.001), and Model 2 (OR: 1.558 [1.257, 1.931], *p* < 0.001). In the fully adjusted model, the highest miR-210 tertile conferred 4.58-fold increased odds of good CCC compared to the lowest tertile (OR: 4.580 [2.088, 10.043], *p* < 0.001). EphrinA3 exhibited a consistent negative association with good CCC formation: Crude Model (OR: 0.995 [0.992, 0.998], *p* < 0.001), Model 1 (OR: 0.995 [0.992, 0.998], *p* < 0.001), and Model 2 (OR: 0.993 [0.990, 0.997], *p* < 0.001). The highest EphrinA3 tertile was associated with 79.4% reduced odds of good CCC compared to the lowest tertile (OR: 0.206 [0.095, 0.446], *p* < 0.001).

### Mediation effect of miR-210 on the association between VEGF-A, EphrinA3 and good CCC

Mediation analysis revealed that miR-210 significantly mediated the relationship between VEGF-A and good CCC (mediation effect: 0.059, 95% CI: 0.026, 0.097, *p* = 0.002), accounting for 77.18% of the total effect (Table 4). Similarly, miR-210 mediated 49.90% of EphrinA3’s influence on good CCC (mediation effect: −0.059, 95% CI: −0.101, −0.027, *p* < 0.001) (Table 5).

**Table 4:** Mediation effect of miR-210 on the association between VEGF-A and good CCC

Mediator	Total Effect	Mediation Effect	Direct Effect	Propotion Mediated(%)
miR-210	0.076 (0.004, 0.151) *p* = 0.036	0.059 (0.026, 0.097) *p* = 0.002	0.017 (−0.061, 0.097) *p* = 0.640	77.18 (20.38, 372.95) *p* = 0.038

Exposure: VEGF-A, Outcome: Good CCC, Adjust: None

**Table 5:** Mediation effect of miR-210 on the association between EphrinA3 and good CCC

Mediator	Total Effect	Mediation Effect	Direct Effect	Propotion Mediated(%)
miR-210	−0.118 (−0.202, −0.038) *p* < 0.001	−0.059 (−0.101, −0.027) *p* < 0.001	−0.059 (−0.153, 0.025) *p* = 0.184	49.95 (19.70, 176.70) *p* < 0.001

Exposure: EphrinA3; Outcome: Good CCC; Adjust: None

## Discussion

CCC serves as a physiological adaptive mechanism responding to significant coronary stenosis or myocardial ischemia in patients with CHD [1]. Our study provides novel insights into the molecular biomarkers associated with CCC development, focusing on miR-210, VEGF-A, and EphrinA3. Our main findings demonstrate that patients with good CCC exhibited significantly higher plasma miR-210 and VEGF-A levels but lower EphrinA3 levels compared to those with poor CCC. Notably, miR-210 showed the strongest association with good CCC formation (highest tertile: 4.58-fold increased odds), while EphrinA3’s highest tertile was associated with 79.4% reduced odds of good CCC. Importantly, mediation analysis revealed that miR-210 served as a key intermediary, mediating 77.18% of VEGF-A’s effect and 49.90% of EphrinA3’s influence on CCC formation.

The extent of coronary stenosis is considered the primary factor influencing CCC development, with stenosis severity correlating with the degree of myocardial ischemia and hypoxia. In our study, patients with good CCC demonstrated elevated Gensini scores, indicating more severe coronary stenosis and consequently more profound hypoxia. This observation aligns with previous findings showing abnormally rich coronary collaterals in conditions associated with hypoxia, such as sleep apnea [23] and high-altitude residence [24]. Experimental studies by Zhang et al. [25] and Aghajanian et al. [26] have confirmed that hypoxia alone can induce new collateral vessel formation in both brain and heart tissues without occlusion, underscoring hypoxia’s fundamental role in collateralization.

In hypoxic conditions, miR-210 emerges as a predominant hypoxia-inducible miRNA [31, 32], being markedly upregulated [33, 34] and functioning as a regulator of the cellular hypoxic response. Our study establishes a significant association between elevated miR-210 expression and good CCC in CHD patients. After adjusting for confounding variables, plasma miR-210 demonstrated strong predictive value for good CCC development across all subgroups, suggesting its potential as a biomarker for CCC formation. This finding extends previous work by Zaccagnini et al. [33, 34], which demonstrated miR-210’s role in peripheral arterial disease models, where its inhibition exacerbated tissue damage and decreased vascular density.

The pro-angiogenic effects of miR-210 appear to operate through multiple mechanisms. Previous studies have established its role in promoting endothelial cell survival, migration, and angiogenic capacity [38–42]. Our findings revealed two potentially critical pathways involving VEGF-A and EphrinA3. First, we observed a positive correlation between miR-210 and VEGF-A levels, consistent with studies by Liu et al. [38] and Hua et al. [35] demonstrating that miR-210 can modulate VEGF-A expression during hypoxia-induced angiogenesis. Concurrently, VEGF-A, recognized as the principal angiogenic factor, contributes significantly to collateral circulation development under both physiological and pathological conditions [25, 27–30]. Our study confirmed significantly elevated VEGF-A expression in patients with good CCC, aligning with Balakrishnan and Kumar’s [29] findings on the correlation between serum VEGF-A and collateral formation in acute coronary syndrome. This relationship is further supported by studies from Clayton et al. [28] and Lucitti et al. [27], which established VEGF-A’s regulatory role in native collateral formation and remodeling. Second, we found reduced EphrinA3 expression in CHD patients with good CCC. This aligns with studies by Fasanaro et al. [39], which identified EphrinA3 as a target of miR-210 in endothelial cells, while members of the Ephrin family act as signaling molecules that modulate angiogenesis within the VEGF signaling pathway [48, 49]. Xiao et al. [42] demonstrated that inhibition of miR-210 upregulates EphrinA3, thereby suppressing angiogenesis. Our data showed EphrinA3 levels exhibited an inverse relationship with both miR-210 and VEGF-A, and as EphrinA3 tertiles increased, the proportion of good CCC decreased. Crucially, our mediation analysis provided insights into the relationships among these molecules, revealing that miR-210 mediates the majority (77.18%) of VEGF-A’s effect and approximately half (49.90%) of EphrinA3’s influence on CCC formation. This suggests that while both VEGF-A and EphrinA3 influence CCC development, miR-210 appears to function as a central regulator in these pathways, coordinating the cellular response to hypoxia in coronary collateralization.

The therapeutic potential of miR-210 has been documented in several preclinical studies [40, 41, 50, 51]. Hu et al. [41] and Arif et al. [40] demonstrated that miR-210 delivery promotes cardiac repair post-myocardial infarction in animal models. Wang et al. [43] showed that mesenchymal stem cell-derived extracellular vesicles carrying miR-210 improved cardiac function through enhanced angiogenesis. These findings, together with our results, suggest that targeted delivery of miR-210 might represent a therapeutic strategy to enhance CCC formation in humans with CHD, though further investigation is required.

This study has several limitations, including its small sample size, single-center design, and observational nature, which precludes establishing causality. Additionally, utilizing the collateral flow index for evaluating CCC might have provided a more precise assessment than the Rentrop system used in this study, as suggested by Seiler et al. [4] in their evaluation of human coronary collaterals.

## Conclusion

In conclusion, plasma miR-210 levels exhibit a strong association with coronary collateral circulation development and represent a promising biomarker for CCC formation in patients with severe coronary stenosis. The influence of both VEGF-A and EphrinA3 on CCC formation appears to be predominantly mediated through miR-210, highlighting its central role in coronary collateralization pathways. Additional investigations are required to clarify the precise mechanisms through which miR-210 influences coronary collateral development and to explore its potential as a therapeutic target for enhancing collateralization in patients with CHD.

## Supplementary Information

Supplementary Material 1.

## Data Availability

No datasets were generated or analysed during the current study.

## Abbreviations

AUC: Area under the curve
BMI: Body mass index
CCC: Coronary collateral circulation
CHD: Coronary heart disease
EC: Endothelial cells
eGFR: Estimated glomerular filtration rate
ELISA: Enzyme-linked Immunosorbent Assay
hypoxamiR: Hypoxia-inducible miRNA
miR-210: MicroRNA-210
miRNA: Micro RNA
OR: Odds ratio
PCI: Percutaneous coronary intervention
qPCR: Quantitative polymerase chain reaction
qRT-PCR: Quantitative Reverse Transcription Polymerase Chain Reaction
ROC: Receiver operating characteristic
VEGF-A: Vascular endothelial growth factor A
