# Supplementary material for: Plasma microRNA-210 is associated with VEGF-A and EphrinA3 and relates to coronary collateral circulation in patients with coronary heart disease: a cross-sectional study
Source: BMC Cardiovasc Disord. 2025 Jul 28;25:547. doi: 10.1186/s12872-025-05013-y (PMC12302828; doi:10.1186/s12872-025-05013-y)

**Plasma microRNA-210 is associated with VEGF-A and EphrinA3 and relates to coronary collateral circulation in patients with coronary heart disease: a cross-sectional study**

*Ning Zhao^2,1^, Kun Na^1^, Wei Sun^2^, Henghe Shi^2^, Xiaolin Zhang^#,1^, Bin Liu^#,^**^2^ and Yaling Han ^#,1, 2^*

*1: State Key Laboratory of Frigid Zone Cardiovascular Disease, Cardiovascular Research Institute and Department of Cardiology, General Hospital of Northern Theater Command, Shenyang, China*

*2: Department of Cardiology, Second Norman Bethune Hospital of Jilin University, No. 218 Ziqiang Street, Changchun, China.*

**Corresponding author**:

Author name: Prof. Yaling Han, Prof. Bin Liu and Prof. Xiaolin Zhang

Mailing address: State Key Laboratory of Frigid Zone Cardiovascular Disease, Cardiovascular Research Institute and Department of Cardiology, General Hospital of Northern Theater Command, Shenyang, China

Tel: +86-24-28856123

Fax: +86-24-28897311

E-mail: hanyaling@163.net, lbin99@jlu.edu.cn, xiaolindianyu75@163.com

**Supplementary Figure Legends**

**Figure S1** **The correlation of BMI, the serum levels of Fasting blood glucose, HDL cholesterol, uric acid and the plasma levels of VEGF-A, miR-210, EphrinA3 and CCC Groups in patients with CHD.**

* p < 0.05; ** p < 0.01; *** p < 0.001. CCC, coronary collateral circulation; BMI, body mass index; HDL, high-density lipoprotein; CHD, coronary heart disease.

**Figure S2 Diagnostic value of plasma miR-210 in patient subgroup analysis.**

eGFR, estimated glomerular filtration rate; OR, odds ratio; CI, confidence interval.

**Supplementary Tables**

**Table S1. Gensini Score Criteria**

| Coronary Artery Stenosis Degree (%) | Score | Lesion Location | Coefficient |
| --- | --- | --- | --- |
| ≤25 | 1 | Left main coronary artery lesion | × 5 |
| ＞25～≤50 | 2 | Proximal left anterior descending branch or Proximal left circumflex branch | × 2.5 |
| ＞50～≤75 | 4 | Mid left anterior descending branch | × 1.5 |
| ＞75～≤90 | 8 | Distal left anterior descending branch or First diagonal branch or Distal left circumflex branch | × 1 |
| ＞90～≤99 | 16 | Proximal, mid, distal right coronary artery and posterior descending branch all | × 1 |
| 100 | 32 | Second diagonal branch or Posterior lateral branch | × 0.5 |

Gensini score= the sum of the scores for each affected vessel, which represents the total score for the patient's coronary artery stenosis.

**Table S2. Primer sequences of miRNA for qRT-PCR**

| Gene name | Forward primer sequence (5′-3′) |
| --- | --- |
| miR-210 | TACTGTGCGTGTGACAGCGGC |
| cel-miR-39 | TCACCGGUGUAAATCAGCTTG |

**Supplementary Figures**


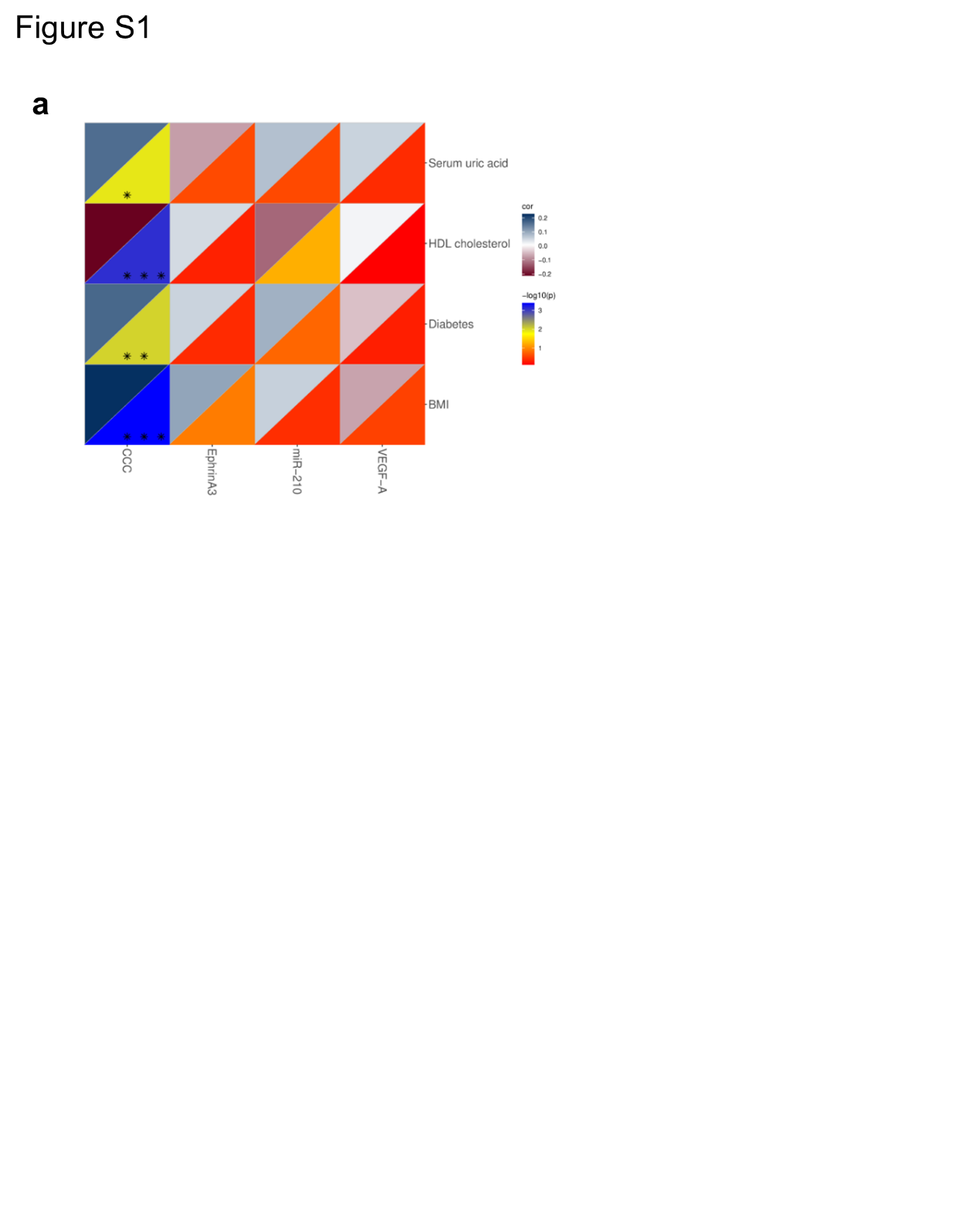


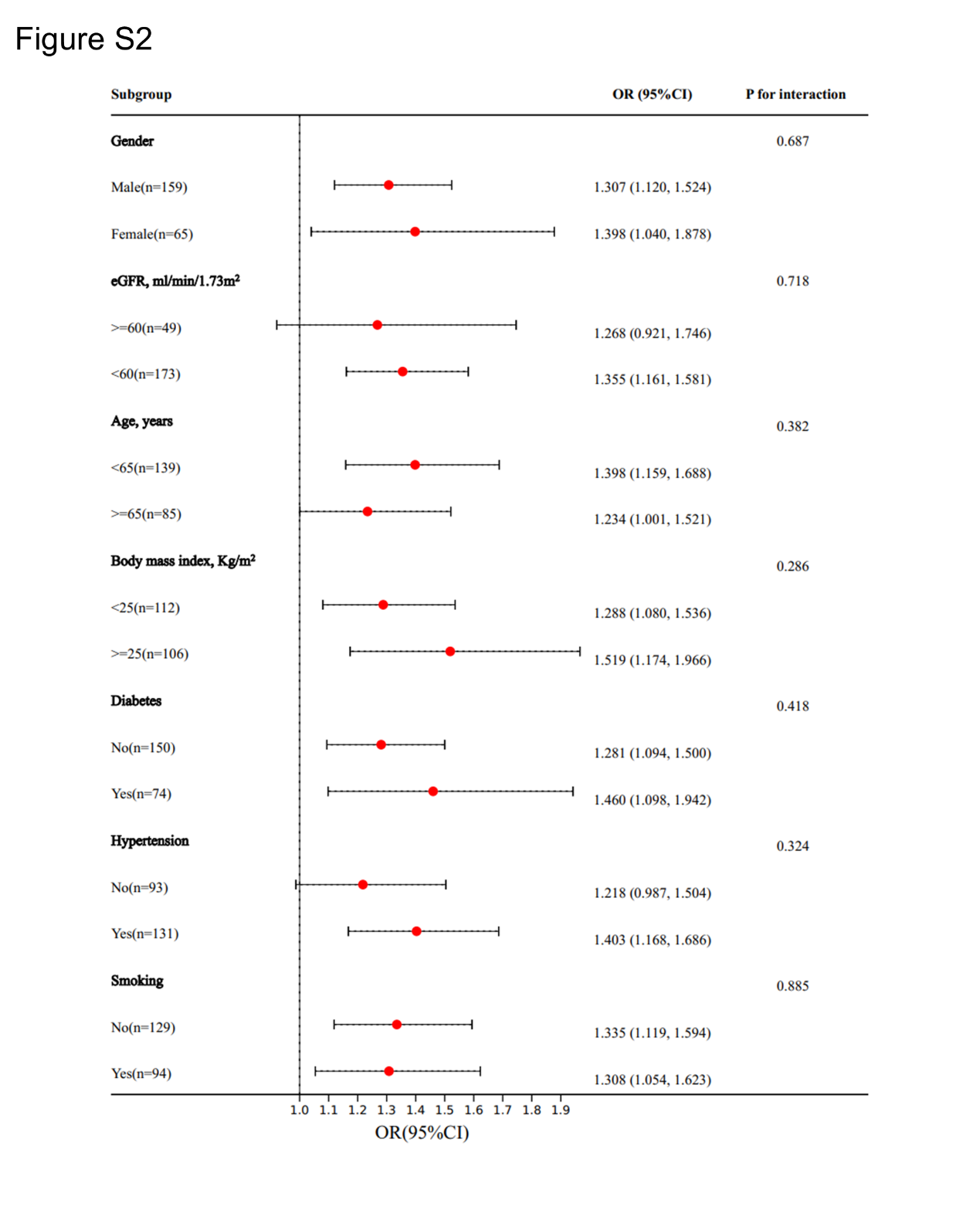

Supplement: Supplementary file 1 — Supplementary Material 1. [file 12872_2025_5013_MOESM1_ESM.docx]
